# Diabetic Retinopathy: Vascular and Inflammatory Disease

**DOI:** 10.1155/2015/582060

**Published:** 2015-06-07

**Authors:** F. Semeraro, A. Cancarini, R. dell'Omo, S. Rezzola, M. R. Romano, C. Costagliola

**Affiliations:** ^1^Department of Medical and Surgical Specialties, Radiological Sciences and Public Health, University of Brescia, Brescia, Italy; ^2^Department of Medicine and Health Sciences, University of Molise, Campobasso, Italy; ^3^Department of Molecular and Translational Medicine, University of Brescia, Brescia, Italy; ^4^Department of Neuroscience, Reproductive Sciences and Dentistry, University of Naples, Italy; ^5^ICRRS Neuromed, Pozzilli, Isernia, Italy

## Abstract

Diabetic retinopathy (DR) is the leading cause of visual impairment in the working-age population of the Western world. The pathogenesis of DR is complex and several vascular, inflammatory, and neuronal mechanisms are involved. Inflammation mediates structural and molecular alterations associated with DR. However, the molecular mechanisms underlying the inflammatory pathways associated with DR are not completely characterized. Previous studies indicate that tissue hypoxia and dysregulation of immune responses associated with diabetes mellitus can induce increased expression of numerous vitreous mediators responsible for DR development. Thus, analysis of vitreous humor obtained from diabetic patients has made it possible to identify some of the mediators (cytokines, chemokines, and other factors) responsible for DR pathogenesis. Further studies are needed to better understand the relationship between inflammation and DR. Herein the main vitreous-related factors triggering the occurrence of retinal complication in diabetes are highlighted.

## 1. Introduction

Vascular complications of diabetes (DM) are classically divided into microvascular (caused by damage to small blood vessels) and macrovascular (caused by damage to larger blood vessels). Microvascular complications include retinopathy, nephropathy, and neuropathy. Diabetic retinopathy (DR) is the most common microvascular complication of DM and a recent report on the prevalence of DR indicated that it is the primary cause of blindness in the working-age population of the Western world [1].

The decrease of retinal perfusion caused by the constriction of major arteries and arterioles is one of the earliest abnormalities observed in DR [2]. This reduction diminishes the retinal blood supply and induces a series of biochemical and metabolic alterations. Retinal pericyte loss is another characteristic feature of DR causing endothelial cell degeneration, microvascular destabilization, and perfusion alterations [3]. Systemic and local hypertension, together with the progressive thickening of the basement membrane, may disrupt the tight link between pericytes and endothelial cells, causing pericyte apoptosis; disruption of proliferation control in the endothelium can give rise to new vessels [4]. These vascular abnormalities result in retinal ischemia, with a release of proangiogenic factors that leads to the development of many abnormal new vessels that proliferate and coat the cortical vitreous surface. Enhanced expression of VEGF attributed to hypoxia and secretion of various proinflammatory cytokines (TNF-*α*, IL-6, and IL-1*β*) are other major alterations observed during progression of DR [5, 6]. Furthermore, in hyperglycemic conditions, retinal mitochondria become dysfunctional and levels of superoxide species increase, which eventually accelerates cytochrome c release, capillary cell apoptosis, and DNA damage [7].

Thus, various interconnecting biochemical pathways have been suggested as potential links between hyperglycemia and DR. These pathways include increased polyol pathway flux [8]; increased expression of growth factors such as vascular endothelial growth factor (VEGF), insulin-like growth factor-1 (IGF-1) [9], tumor necrosis factor-alpha (TNF-alpha) [10], erythropoietin [11, 12], and adiponectin [13]; hemodynamic changes; accelerated formation of advanced glycation end products (AGEs); oxidative stress; activation of the renin-angiotensin-aldosterone system (RAAS); and subclinical inflammation and leukostasis [14].

Neuroinflammation also plays an important role in the pathogenesis of DR. It has been proposed that, in neuroinflammation, also known as “microglial activation” or neural “pseudo-inflammation,” microglia become activated and produce inflammatory mediators. In fact, neuroinflammation does not mean microglial activation; microglial activation is the main mechanism by which neuroinflammation starts in response to nervous tissue perturbations [15].

**Figure 1:**
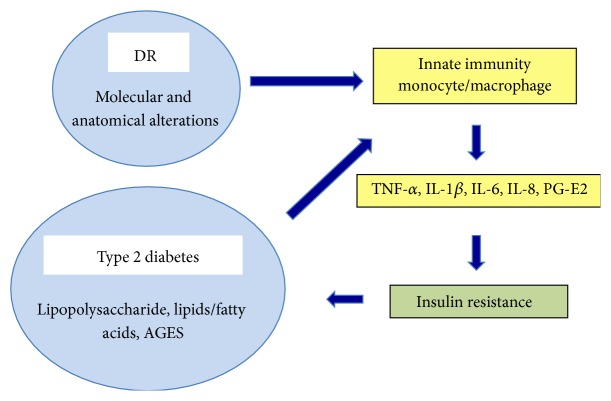
The role of innate immunity in diabetic retinopathy and type 2 diabetes mellitus. In patients with type 2 diabetes and diabetic retinopathy, innate immune markers and proinflammatory cytokines, including IL-1*β*, IL-6, IL-8, TNF-*α*, and prostaglandin E2, are upregulated. The cytokines then enter systemic circulation and contribute to the diabetic pathology by increasing insulin resistance and by elevating blood glucose levels.

Recent studies have shown that the expression of several cytokines, chemokines, and other factors is increased in diabetes and that the levels of these molecules are particularly elevated in the ocular fluid of diabetic patients (Figure 1) [5, 6, 15–23].

This review highlights the role of inflammation in DR development and progression by describing the inflammatory mediators involved in DR pathogenesis (Table 1).

## 2. Inflammation and DR

The involvement of inflammatory processes in inducing structural and molecular alterations associated with DR is gaining increasing attention, and it has been particularly associated with the early stages of DR. However, the underlying molecular mechanisms have not been clearly characterized [16]. At least four distinct biochemical pathways have been associated with the development of DR: (i) increased polyol pathway flux, (ii) increased advanced glycation end products (AGEs) formation, (iii) activation of protein kinase C (PKC) isoforms, and (iv) increased hexosamine pathway flux [24]. Dysregulation of these molecular pathways results in excessive production of free radicals in mitochondria, increased oxidative stress, abnormal rheology, and activation of the renin-angiotensin system, and each of these pathways contributes to upregulation of growth factors and inflammatory cytokines. The resulting alterations decrease vascular wall integrity, increasing vascular permeability, lumen occlusion, and ischemia [25].

Hyperglycemia is the main culprit in DR development; it affects the levels of plasma cytokines (TNF-*α* and IL-6) implicated in insulin resistance [26]. In addition, the high blood glucose levels in diabetic patients can stimulate the production of erythropoietin (EPO) and VEGF [7, 27]. Retinal hypoxia is an important causative factor of DR and leads to the release of many soluble factors in the vitreous, including inflammatory molecules such as cytokines, chemokines, and growth factors [6]. Ischemia is associated with inflammatory manifestations because it induces a signaling pathway that attracts macrophages into hypoxic areas through the expression of chemokines such as monocyte chemotactic protein-1 (MCP-1). Hypoxia-activated macrophages and microglia, the immune cells of the retina, release TNF-*α*, which stimulates the release of IL-8, MCP-1, and VEGF in vascular cells or in retinal microglia cells [28]. Such alterations in the endothelial cells of the retinal vessels lead to retinal ischemia, which stimulates the production of angiogenic factors such as VEGF and EPO [29–31]. DR-related inflammatory responses may be exacerbated by altered retinal fatty acid metabolism, interaction of AGE with RAGE via MAPK pathway, and hypertension-induced increases in VEGF and intercellular adhesion molecule- (ICAM-) 1 expression [16].

## 3. Inflammation and Angiogenesis Cross Talk in DR

Inflammatory cells may produce angiogenic cytokines, growth factors, and proteases that contribute to the formation of new vascular structures, tissue damage, or tumor growth at the site of inflammation [32]. Microvascular endothelium activated by a number of cytokines and angiogenic growth factors can express proinflammatory molecules involved in leukocyte recruitment and activation [33–35]. Neovascularization and inflammation share a number of common mediators and signaling pathways such as the cyclooxygenase (COX)/prostaglandin pathway [36]. Various chemokines may act both as leukocyte attractants and as angiogenic inducers by acting directly on endothelial cells [37]. In addition, a number of proinflammatory cytokines (e.g., IL-1*α*, IL-1*β*, IL-6, TNF-*α*, high-mobility group box-1 (HMGB1), and osteopontin) may induce blood vessel formation via direct engagement of target endothelial cells or, indirectly, by inducing leukocytes and/or endothelial cells to produce proangiogenic mediators [38–40]. Conversely, the angiogenic factors VEGF and angiopoietin-1 may elicit proinflammatory responses in endothelial cells by upregulating the expression of cell adhesion molecules and inflammatory mediators [41, 42].

The role of inflammation and angiogenesis in the pathogenesis of DR is widely accepted [5, 21]; however, some of the pathogenic and molecular aspects still need to be carefully investigated. Several inflammatory molecules including ICAM-1, TNF-*α*, IL-1, and COX-2 released by activated inflammatory cells and glial elements play a major role in the degeneration of retinal capillaries [43]. Inflammatory cytokines enhance leukocyte adhesion to endothelium, vascular permeability, and thrombus formation by inducing procoagulant activity and by inhibiting anticoagulant activity [44]. Leukocytes attach to the vascular epithelium and then release inflammatory cytokines, growth cytokines, and vascular permeability factors, which alter endothelial junction proteins, enable leukocyte diapedesis into the retina, and compromise the blood-retinal barrier (BRB) [45]. The inflammatory responses may contribute to neovascularization, especially under hypoxic conditions. Inhibition of leukocyte adhesion, depletion of phagocytic cells [46], and deletion of the receptors for MCP-1, ICAM-1, or CD-18 all inhibit pathological neovascularization. Inhibition of COX causes a reduction in the production of prostanoid, which in turn reduces retinal neovascularization and lowers the production of VEGF [47].

## 4. Inflammation and DME

DME is the major cause of loss of vision and blindness in people with DR [1]. DME results from the intraretinal accumulation of fluid due to the breakdown of the BRB and the leakage from microaneurysm, capillaries, and arterioles. The pathogenesis of DME is a complex pathological process with multiple contributing factors that lead to the dysfunction of the inner and outer retinal barriers and to the accumulation of sub- and intraretinal fluid in the inner- and outer-plexiform layers. The BRB is formed by an extensive junction complex between retinal pigment epithelial cells and vascular endothelial cells. The breakdown of BRB results in accumulation of plasma proteins that exert a high oncotic pressure in the neural interstitium, which tends to produce interstitial edema. Several factors, including hyperglycemia, hypertension, high cholesterol, VEGF, hypoxia, ischemia, oxygen-free radicals, AGE, PKC, inflammatory mediators and Muller cells, pericyte, and glial cell dysfunction, are all involved in the breakdown of BRB [48]. One of the first major events in the BRB dysfunction is leukostasis, which involves the accumulation of leukocytes on the luminal surface of the retinal capillaries. AGEs upregulate NF-*κ*B in the retinal microvascular endothelium and an AGE-dependent increase in leukocyte adhesion* in vitro* has been observed; increased leukocyte adhesion* in vivo* was accompanied by blood-retinal barrier dysfunction [49]. Adherent leukocytes mediate vascular leakage [50]. Kaji et al. demonstrated in a murine model that interaction of AGEs with RAGE leads to leukostasis and blood-retinal barrier breakdown [51]. Leukostasis contributes to DR through the upregulation of retinal ICAM-1 and CD-18 and through tight junction dysfunction and disorganization. Leukocyte adhesion to the diabetic vascular endothelium can promote receptor-mediated endothelial apoptosis via Fas/FasL, as shown in a short-term model of diabetic retinopathy [50]. Inhibition of leukocyte adhesion also prevented the loss of pericytes, which are not typically in direct contact with adherent leukocytes in the vasculature [50]. Finally, leukocytes produce reactive oxygen species and inflammatory cytokines, leading to increased vascular permeability. Inflammatory cytokines induce VEGF-A activation, which in turns leads to the destruction of BRB [16, 52].

Recently, Noma et al. [53] measured the vitreous levels of VEGF, soluble VEGF receptor, soluble ICAM-1, MCP-1, and pentraxin 3 in patients with DME and in healthy controls. Vitreous fluid levels of these cytokines were significantly higher in the patients with DME than in controls, indicating that in DME patients inflammatory factors may induce an increase in vascular permeability with a consequent disruption of the blood-aqueous barrier. Whether increased permeability causes retinal inflammation in diabetes or inflammatory changes cause the diabetes-induced increase in permeability or both has not yet been adequately addressed.

## 5. Vitreous Mediators of DR

Under hypoxic-ischemic conditions, the retinal tissue induces glycolysis, angiogenesis, vasodilation, and erythropoiesis to compensate for the oxygen deficit. Under hypoxic conditions, a variety of soluble factors, including cytokines, chemokines, and growth factors, are secreted into the vitreous cavity [6, 54]. However, the mechanisms to protect against hypoxia-related damage are lost within hours of the hypoxic-ischemic insult and cell death and tissue damage eventually ensues.

**Figure 2:**
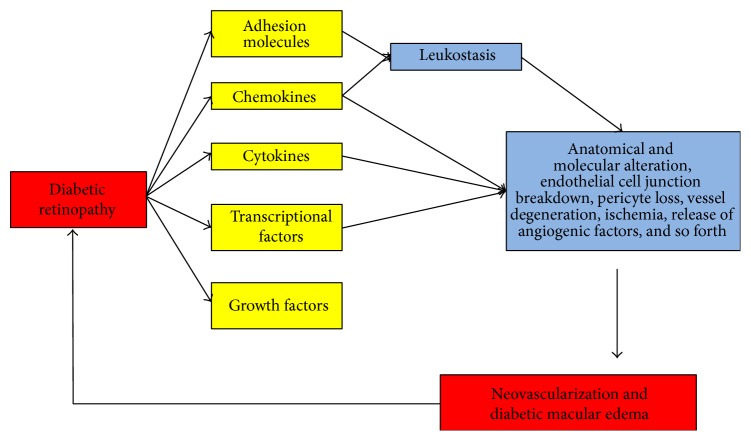
Role of vitreous mediators in DR progression. In DR, several inflammatory vitreous mediators are upregulated and induce anatomical changes in the retinal tissue. The structural changes enhance retinal tissue degeneration and mediate pathogenesis of DR.

Diabetes mellitus can lead to retinal tissue hypoxia and dysregulation of immune responses in the retinal tissue. To identify the immune mediators involved in DR pathogenesis, vitreous samples obtained from DR patients have been analyzed. When BRB is functional, cytokines and growth factors in the vitreous are locally produced [54]. Numerous studies have demonstrated an association between the severity of DR and intravitreal levels of cytokines, chemokines, growth factors, and extracellular matrix proteins; all of these compounds are able to maintain an inflamed environment, further stimulating endothelial cell secretion and neovascularization [16–20, 54]. Cytokine and chemokine levels are also increased in the epiretinal membranes of patients with DR [55], and an association between the levels of these molecules and disease severity has been demonstrated [54]. The primary vitreous-related factors involved in the pathogenesis of DR and their role in DR pathogenesis are summarized in Table 2 and Figure 2. Several studies have found increased intraocular levels of some cytokines in patients with DR [19, 20]. The roles of cytokines, chemokines, transcription factors, and other immune mediators in the development of DR are summarized in the following paragraphs of this section.

## 6. Growth Factors

VEGF plays a critical role in both vasculogenesis and angiogenesis [56–59]. VEGF increases vascular permeability, stimulates angiogenesis because of its mitogenic effect on endothelial cells, and enhances endothelial cell migration and survival [60, 61]. Under hypoxic conditions, a 3- to 12-fold increase in VEGF expression has been reported [62, 63]. VEGF enhances the adhesion of leukocytes to vascular walls and increases ICAM-1 and vascular cell adhesion molecule-1 (VCAM-1) expression in the brain and retina [64]. VEGF also promotes the expression of ICAM-1 in endothelial cells, and the elevated ICAM-1 expression leads to activation of leukocytes and cytokine production. The cytokines in turn mediate the inflammatory response and stimulate further release of  VEGF. Inhibition of VEGF produced in Muller cells in diabetic mice reduces the expression of TNF-*α*, ICAM-1, and NF-*κ*B [65]. Several reports indicate that VEGF is a proangiogenic cytokine [66–68]. Under hypoxic conditions, VEGF production is increased and the permeability of BRB is enhanced. The role of VEGF in BRB permeability has been further corroborated by the finding that melatonin-mediated inhibition of VEGF production reduces BRB permeability [69]. Thus, VEGF is believed to be the main angiogenic growth factor responsible for the development of DR [70]. Several studies have shown a strong correlation between increased levels of intravitreal VEGF and the development of DR [67, 71–73]. Additionally, a correlation between VEGF levels and retinopathy activity has also been demonstrated [74]. VEGF and its receptor are also located on the epiretinal membrane in the eyes of patients with diabetes [73].

Placental growth factor (PGF) is a member of the VEGF subfamily and is involved in angiogenesis. PGF can increase the activity of low concentrations of VEGF and indirectly stimulate endothelial cell proliferation, migration, and angiogenesis [75, 76]. Increased levels of intravitreal PGF are observed during the course of PDR, and these levels are significantly correlated with VEGF levels [74, 77, 78]. Therefore, PGF appears to be involved in the pathogenesis of DR.

Tenascin-C is an extracellular matrix glycoprotein that modulates cell growth and cell adhesion. It is expressed in developing organs and during oncogenesis, wound repair, and inflammation [79]. Tenascin-C is also involved in the sprouting of endothelial cells as the first necessary step in angiogenesis [80]. Compared to healthy controls, diabetic patients have increased vitreous tenascin-C levels. In addition, intravitreal levels of tenascin-C are higher in patients with active PDR than in those with quiescent PDR [81]. Furthermore, tenascin-C has been identified in the epiretinal membrane of patients with PDR [82, 83]. Therefore, it appears likely that tenascin-C is also involved in the pathogenesis of PDR.

Insulin-like growth factor-1 (IGF-1) is a polypeptide that regulates the proliferation and differentiation of several cell types [84]. In human eyes, IGF-1 is produced by retinal pigment epithelium cells, pericytes, and endothelial cells, and experimental studies indicate that IGF-1 stimulates the production of VEGF [85]. Levels of intravitreal IGF-1 were found to be significantly increased in the eyes of patients with PDR compared to those of controls [84–86].

Basic fibroblast growth factor (bFGF) is a growth factor that is important for the survival and maturation of neurons and glial cells. bFGF plays an important role in tissue repair and is also an angiogenic factor [87, 88]. bFGF and its receptor are expressed in the retina, and its concentration in the vitreous is increased in patients with PDR [86, 89]. bFGF is therefore involved in the pathogenesis of PDR [54] and seems to stimulate VEGF production [6]. It is also involved in the formation of epiretinal membranes [90]. bFGF and its receptor are expressed in the retina, and its concentration in the vitreous is increased in patients with PDR [86, 89]. Furthermore, glial cell line-derived neurotrophic factor stimulates Muller cells to produce bFGF [91, 92], which in turn stimulates endothelial cell proliferation and secretion of VEGF [93]. In addition, studies have shown that bFGF, nerve growth factor, and glial cell line-derived neurotrophic factor are involved in the formation of epiretinal membranes in PDR [54]. Taken together, these studies indicate that bFGF is involved in the pathogenesis of PDR.

Hepatocyte growth factor (HGF) and its receptor modulate the motility, growth, and morphogenesis of various cell types [94] and have been shown to have angiogenic activity. Intravitreal HGF levels of PDR patients are significantly higher than those of controls [95]. Furthermore, aqueous HGF levels are positively correlated with the degree of DR [96].

Connective tissue growth factor (CTGF) is involved in the stimulation of proliferation, angiogenesis, migration, extracellular matrix production, cell attachment, cell survival, and apoptosis [97]. Both CTGF and VEGF levels are elevated in the vitreous of PDR patients. CTGF may promote the formation of proliferative membranes in PDR. CTGF may indirectly modulate VEGF expression but has no effect on retinal neovascularization [98].

Stem cell factor is important for the survival and differentiation of hematopoietic stem cells. Several studies have demonstrated that stem cell factor signaling promotes the survival, migration, differentiation, and capillary tube formation of endothelial cells and plays an important role in ischemia-induced neovascularization [99].

EPO is a multifunctional glycoprotein that is mainly produced in the fetal liver and adult kidney in response to hypoxia [100] and has antioxidant, anti-inflammatory, proangiogenic [101–105], neuroprotective [106], and antiapoptotic properties [107–109]. Recent studies have shown that production of EPO also takes place in the retina in response to hypoxic stimuli [101, 109, 110] and that its expression is mediated by HIF-1*α*, which also stimulates the secretion of VEGF [109–111]. Increased expression of EPO in both the retinal pigment epithelium and neuroretina has been found in the eyes of patients with DR [98]. Through an autocrine/paracrine mechanism of action, locally produced EPO preserves the integrity of the outer retina barrier [103, 109, 110] and protects pericytes through its antioxidant and anti-inflammatory activity. Furthermore, EPO prevents glucose- and free radical-induced apoptosis of retinal cells [112–114]. However, EPO also has the undesirable property of potently stimulating neovascularization [101, 115]. The interactions between VEGF and EPO in the eyes of patients with PDR have not been fully elucidated. EPO and VEGF are both upregulated in the vitreous of patients with PDR and they appear to act independently [12, 13, 101]. Levels of EPO are higher than VEGF, and EPO inhibition suppresses retinal neovascularization both* in vivo* and* in vitro*. Suppression of EPO and VEGF leads to an inhibition of retinal neovascularization greater than that achieved by each compound alone.* In vitro* inhibition of EPO leads to attenuation of endothelial cell proliferation in PDR [116]. In murine models of oxygen-induced retinopathy, inhibition of EPO led to inhibition of retinal neovascularization* in vivo* and inhibition of retinal endothelial cell proliferation* in vitro* [101]. Although it is tempting to use EPO as a target for novel retinal antiangiogenic treatments, its neuroprotective effects on retinal cells must first be determined [106].

Aminopeptidase N (APN) is a polypeptide hormone that is exclusively produced in adipocytes and circulates at very high levels in the bloodstream. In experimental studies, APN has been shown to exert both anti-inflammatory and antiatherosclerotic effects, as well as to inhibit neointimal thickening and vascular smooth muscle cell proliferation in mechanically injured arteries [117]. Low plasma APN concentrations are associated with obesity, insulin resistance, type 2 diabetes, coronary disease, and hypertension [118]. Several studies have indicated that APN possesses anti-inflammatory properties, which may negatively modulate atherogenesis [119]. The role of APN in the development of microvascular disease is largely unknown. Clinical data indicate that the APN levels in patients with both type 2 diabetes and DR are lower than in diabetic patients without DR [120]. Costagliola et al. demonstrated that APN levels in aqueous humor of patients with type 2 diabetes, PDR, and macular edema are higher than in aqueous of control subjects [13].

## 7. Cytokines

IL-6 is a multifunctional cytokine involved in regulating immune responses, increasing vascular permeability, and stimulating angiogenesis [121, 122]. In addition, IL-6 regulates the expression of matrix metalloproteinases (MMPs) such as MMP-1, MMP-2, MMP-3, MMP-9, and MMP-13. MMPs are important regulators of extracellular matrix, which is the primary component of the vitreous [123, 124].* In vitro* studies have indicated that IL-6 increases the permeability of endothelial cells and thereby induces actin filament rearrangement and alters the shape of endothelial cells [20]. Analysis of vitreous IL-6 and IL-8 levels indicates that the levels of both cytokines are higher in diabetic patients than in nondiabetic patients [5, 17–20, 125–128]; however, the precise role of these cytokines in DR pathogenesis has not been fully characterized. We speculate that the angiogenic and chemoattractive properties of IL-8 may mediate inflammatory responses in the retina. Several studies have shown that vitreous IL-6 levels are correlated with the severity of PDR, neovascularization, and macular thickness [5, 19, 127–132]. The prominent role of IL-6 in neovascularization, a key clinical feature of DR, is further highlighted by previous studies that show that IL-6 can not only promote angiogenesis directly but also support angiogenesis by inducing expression of VEGF [19], a potent angiogenic factor. Therefore, IL-6 and IL-8 may be major mediators of retinal inflammation and neovascularization.

IL-1*β* is an inflammatory cytokine mainly produced by macrophages and it can activate the transcriptional factor nuclear factor-kappa B (NF-*κ*B), which is involved in the transcription of inflammatory cytokines [132]. Recombinant IL-1*β* along with TNF-*α* has been shown to stimulate human retinal pigment epithelium cells to secrete IL-6 and IL-8 [133]. Furthermore, IL-1*β* together with TNF-*α* promotes angiogenic activity. IL-1*β* stimulates the synthesis of collagen, glial cells, and fibroblasts, causing proliferation and contraction.* In vivo* studies have demonstrated that IL-1*β* promotes angiogenesis and mediates ocular neovascularization [134]. The role of IL-1*β* in the pathogenesis of DR has recently been studied using diabetic mice with inhibited IL-1*β* activity either by suppressing an enzyme required for the production of IL-1*β* or by blocking the receptor for IL-1*β*. The mice with functional IL-1*β* signaling pathway exhibited clinical pathology of DR, which was significantly lower in mice with inhibited IL-1*β* pathway. Thus, IL-1*β* plays an important role in the development of diabetes-induced retinal diseases [135]. Animal studies indicate that the levels of IL-1*β* are increased in the retina of diabetic rats and that intravitreal injection of IL-1*β* or exposure of retinal endothelial cells to this cytokine causes degeneration of the retinal capillary endothelial cells [136]. In contrast to the findings of the animal studies, few studies with human subjects have found increased levels of IL-1*β* in the vitreous of diabetic patients as compared to that of nondiabetic patients [17]. This discrepancy may have resulted from the type of IL-1*β* detection assays used.

TNF-*α* is a cytokine synthesized by macrophages and T cells and its expression is regulated by NF-*κ*B [137]; it is an inflammatory mediator of neuronal death after ischemic injury in the brain and retina [138]. TNF-*α* was identified by studying its antiangiogenic activity; when injected into tumors, it causes tumor vessel regression, resulting in tumor necrosis [139]. However, it may also have proangiogenic effects under certain conditions. TNF-*α* recruits inflammatory cells, which stimulate neovascularization in some circumstances and inhibit it in others. Therefore, the effect of TNF-*α* in various tissues and disease processes is difficult to predict and must be determined by experimentation [6]. TNF-*α* increases retinal endothelial permeability by downregulating the expression of tight junction proteins and the increased permeability can lead to rupturing of the BRB [140]. TNF-*α* can also stimulate leukocyte adhesion and induce oxidation and production of reactive oxygen species due to the death of retinal ganglion cells and degeneration of the optic nerve [141]. Increased levels of TNF-*α* have been demonstrated in proliferative retinopathies and in animal models of retinal neovascularization. The increased levels of TNF-*α* in the presence of VEGF can stimulate the generation of new retinal vessels. TNF-*α* is also a chemoattractant for leukocytes [142]. TNF-*α* has been associated with the pathogenesis of several chronic inflammatory diseases, including type 2 diabetes [143]. Diabetic patients have higher TNF-*α* levels than nondiabetic patients, and a strong correlation between plasma TNF-*α* levels and the severity of DR was reported [144]. The intraocular production of TNF-*α* is higher than that at the systemic level, and both vitreous TNF-*α* levels and the TNF-*α* vitreous/serum ratios of diabetic patients were found to be higher than those of the nondiabetic patients [17, 144]. Moreover, TNF-*α* is expressed in the endothelial cells and stromal cells of the fibrovascular membranes of diabetic patients with PDR [5]. Recently, Costagliola et al. have found that the TNF-*α* concentration in tears increases with the severity of pathology, the levels being lower in nondiabetic patients than in diabetic subjects, and that the levels were highly correlated with DR severity [10]. The intravitreal injection of an inhibitor of TNF-*α* leads to a significant reduction in the loss of pericytes and capillary degeneration in diabetic mice [145, 146], and TNF-*α*-deficient mice show decreased vascular changes induced by diabetes [147].

HMGB1 is a protein that stabilizes the formation of nucleosomes and gene transcription [148]. It is expressed in numerous sites in the retina, including the ganglion cell layer, inner nuclear layer, outer nuclear layer, inner and outer segment of the photoreceptors, and retinal pigment epithelial cells [149, 150]. Furthermore, HMGB1 can be actively secreted by different cell types, including monocytes and activated macrophages, mature dendritic cells, natural killer cells, and endothelial cells [21]. HMGB1 may play a key role in the protection of retinal injury after ischemia-reperfusion [151]. Therefore, HMGB1 functions as a cytokine or a cofactor that amplifies the effect of the receptor for AGE (RAGE) axis in an autocrine/paracrine manner and mediates the secretion of survival factors, including VEGF-A, to counteract the effects of oxidative stress [6]. At the extracellular level, HMGB1 acts as a proinflammatory cytokine. HMGB1 binds to RAGE and leads to activation of NF-*κ*B, which in turn leads to overexpression of proinflammatory molecules such as TNF-*α*, MCP-1, ICAM-1, and VEGF [152, 153]. Therefore, HMGB1 is thought to contribute to the accelerated micro- and macrovasculopathy observed in diabetes [154]. El-Asrar et al. recently reported that HMGB1 and its receptor RAGE are expressed in the endothelial cells and stromal cells of the epiretinal fibrovascular membranes in PDR, and a significant correlation was found between the expression levels of RAGE and HMGB1 and the levels of neovascularization in PDR with fibrovascular membranes. They also demonstrated the presence of high levels of HMGB1 in the vitreous of patients with PDR [152].

## 8. Transcription Factors

The increased expression of inflammatory molecules is determined by the activation of proinflammatory transcription factors such as NF-*κ*B. NF-*κ*B is an inducible transcription factor and is one of the main regulators of inflammatory immune responses, cell proliferation, and apoptosis [155]. NF-*κ*B is activated in endothelial cells and retinal pericytes in response to hypoxia and hyperglycemia, both* in vitro* and* in vivo*. The activation of NF-*κ*B leads to the synthesis of several cytokines, chemokines, and proinflammatory molecules [16]. Receptor activator of NF-*κ*B (RANKL), a member of the TNF superfamily, is a potent stimulant for the production of proinflammatory molecules through the upregulation of NF-*κ*B upon binding to its ligand RANK. The signal related to RANKL plays a role in the pathogenesis of insulin resistance and suggests a link between inflammation and the pathogenesis of type 2 diabetes mellitus [156, 157].

Hypoxia-inducible factor-1 (HIF-1) is a transcription factor that regulates the cellular response under both acute and chronic hypoxic conditions [158]. Under hypoxic conditions, HIF-1*α* is produced continuously and then degraded, whereas, under conditions of reduced oxygen tension, HIF-1*α* rapidly accumulates and starts to act as a transcription factor in the nucleus, moving and activating several genes, including the genes for IL-6, IL-8, and proangiogenic growth factors [159]. Several researchers have shown that diabetic factors result in HIF-1 production and angiogenesis. Treins et al. showed that insulin-like growth factor-1 (IGF-1) stimulates accumulation of HIF-1 in human retinal pigment epithelial cells [160]. VEGF expression seems to be regulated through two interdependent mechanisms: directly via HIF-1 and indirectly via NF-*κ*B and COX-2 expression and prostaglandin E2 production. Acute intensive insulin therapy exacerbates the diabetic BRB breakdown through HIF-1 and VEGF [161]. This could explain why intensive control of insulin levels can result in transient worsening of DR. Recently, the presence of HIF-1a has been demonstrated in diabetic epiretinal membranes [162]. NF-*κ*B plays a role in regulating the expression of the HIF-1 in response to inflammatory stimuli [163]. Hypoxia induces activation of NF-*κ*B, which, upon binding to the promoter of HIF-1, stimulates the production of IL-6 and IL-8 in the vitreous of patients with PDR [130]. There are conflicting results as to whether or not the activity of NF-*κ*B and HIF-1 increases in the vitreous of patients with PDR [130]. Therefore, to date, the role of NF-*κ*B or HIF-1 in the regulation of the PDR process remains poorly understood.

## 9. Chemokines

MCP-1 is a chemokine that plays an important role in the recruitment of leukocytes and is a powerful stimulus that induces fibrosis and angiogenesis [164]. MCP-1 stimulates the migration of monocytes and macrophages in tissues and is a potent activator of these cell types. The expression of MCP-1 is regulated by NF-*κ*B and MCP-1 can induce VEGF production [165]. Experimental studies have demonstrated an association between macrophage activation and retinal angiogenesis, suggesting that macrophages play an important role in the pathogenesis of DR. Capillary occlusion, which causes retinal ischemia, may be due to the adhesion of macrophages to the capillary endothelium [20, 126]. The presence of macrophages in the secondary epiretinal membranes has also been documented [166]. MCP-1 levels have been found to be elevated in the vitreous of diabetic patients, and the MCP-1 levels are higher in the vitreous than in the serum. The expression pattern indicates that MCP-1 is produced locally [5, 17, 18, 20, 167]. A previous study showed that there was a significant association between the vitreous MCP-1 levels and DR severity [168]. Furthermore, a negative relationship between the levels of MCP-1 and preoperative laser treatment has been demonstrated [151]. MCP-1 has also been shown to be a significant component of the retinal inflammation induced by diabetes [169] and its production is increased in endothelial cells, retinal pigment epithelial cells, and glial cells in patients with hyperglycemia [169]. Furthermore, MCP-1 is expressed in myofibroblasts and in the epiretinal membranes of diabetic patients [170].

Interferon-gamma inducible protein 10 (IP-10) is a CXC chemokine that is expressed at higher levels in the vitreous of diabetic patients than in that of controls [60], and its vitreous levels are higher than its serum levels [18]. IP-10 is a potent inhibitor of angiogenesis* in vivo* [70]. High levels of IP-10 in the vitreous of diabetic patients may counteract the angiogenic effects of VEGF and other proinflammatory cytokines.

Monokine induced by interferon-gamma (MIG) is principally known as a chemoattractant for activated T cells and also has angiostatic activity. Wakabayashi et al. [171] recently documented that the vitreous MIG concentration in DR patients is significantly higher than that in the control subjects. The study also showed that there was a significant correlation between the vitreous concentrations of MIG and VEGF. However, it is currently not clear why MIG, an angiostatic factor, would be elevated in the vitreous of diabetic patients. One possibility is that MIG levels are elevated in response to the upregulation of angiogenic factors such as VEGF. A second hypothesis is that in DR MIG might play a role in the chemotaxis of leukocytes rather than in carrying out its angiostatic functions; leukostasis has been characterized as a pathogenic mechanism of DR [50]. SDF-1 (stromal cell-derived factor-1) is a chemokine that is upregulated in response to tissue damage; it stimulates the mobilization of cells involved in tissue repair as well as the migration, differentiation, and proliferation of endothelial progenitor cells [51]. SDF-1 acts as an angiogenic agent in several model systems [172]. SDF-1 promotes repair after ischemic injury by binding to its receptor, CXCR4, to recruit the progenitors of endothelial cells from the bone marrow. The SDF-1 receptor CXCR4 is expressed by endothelial cells, and its expression increases after treatment with VEGF or basic fibroblast growth factor [172]. SDF-1 also induces the expression of VEGF, thereby stimulating angiogenesis [173]. SDF-1 acts together with VEGF in promoting the recruitment of endothelial progenitor cells from remote sites to the ischemic retina [174]. The concentration of SDF-1 was found to be increased in the vitreous of patients with both DME and PDR, and this increase is correlated with the severity of the disease [175]. Another study found that SDF-1 protein is highly expressed in both the vitreous and preretinal membranes of PDR patients; SDF-1 may be associated with VEGF in angiogenesis in PDR [176]. Increased SDF-1 and VEGF levels in the vitreous were able to induce DR in a mouse model, and these levels were dramatically reduced after intravitreal injection of triamcinolone [103]. These data demonstrate that SDF-1 plays an important role in the development of DR and could be a target for future therapies [21]. Butler et al. showed that one intravitreal injection of a blocking antibody to SDF-1 could block neovascularization in an acute injury model for up to 1 month; they thus suggested that using antibodies to block SDF-1 activity may provide a safe and effective alternative treatment for ischemic diseases, such as PDR and DME [173].

Fractalkine concentrations in vitreous of PDR patients are higher than in control samples, indicating that it may be a potent angiogenic mediator* in vitro* and* in vivo*, and may play an important role in ocular angiogenic disorders such as PDR [175].

Macrophage migration inhibitory factor (MIF) is a chemokine that prevents the random migration of macrophages, stimulates their recruitment at sites of inflammation, and increases their adherence, motility, and phagocytosis [71]. Similar to MCP-1, MIF may be involved in the recruitment of macrophages to the eyes of patients with DR [54]. In support of this hypothesis, Mitamura et al. [177] have found increased intravitreal levels of MIF in the eyes of patients with PDR. Previous studies also have shown a relationship between MIF levels and the clinical stage of DR [168] and they have shown that intravitreal MIF levels were associated with the presence of proliferative epiretinal membranes [177].

## 10. Adhesion Molecules

Activation of RAGE, oxidative stress, vascular leakage in the diabetic retina, capillary nonperfusion, and damage of endothelial cells are all related to increased expression of adhesion molecules that allow the adhesion of leukocytes to the endothelium [16, 21, 50] and expression of retinal vascular adhesion molecules such as VEGF [178]. ICAM-1 is the primary adhesion molecule involved in DR pathogenesis. The levels of ICAM-1 in the vitreous are increased in diabetic patients with PDR and are higher in patients with active PDR than in those with inactive PDR [152]. The expression of ICAM-1 is increased in the retinal vessels of the choroid and in the fibrovascular membranes of diabetic patients and is correlated with the number of neutrophils moved into the retina and choroid, indicating that high ICAM-1 levels facilitate the recruitment of leukocytes [179]. Diabetic mice deficient in ICAM-1 or CD-18 (gene encoding ICAM-1 ligand) show a reduction in the initial lesions associated with DR (such as the loss of pericytes, degeneration, and increased capillary permeability) and leukostasis [50]. Additionally, in murine model, Adamis demonstrated that CD-18 blockade significantly decreases leukostasis in the diabetic retinal microvasculature [180]. E-selectin and VCAM-1 are also involved in the pathogenesis of DR, and their soluble forms were found to be increased in the vitreous of diabetic patients [181, 182]. Both VCAM-1 and E-selectin may act as angiogenic factors in endothelial cells; indeed, there is a direct correlation between VCAM-1 and VEGF levels [181]. Soluble vascular adhesion proteins increased in both the vitreous and serum of patients with PDR [183], and its production is increased in the presence of high levels of glucose and proinflammatory cytokines such as TNF-*α* and IL-1*β* [21]. Therefore, this molecule also seems to be involved in the pathogenesis of DR [21].

## 11. Other Molecules

Several studies have reported an increase in some components of the complement system in the vitreous and/or epiretinal membranes of diabetic patients with PDR, including C3, factor I, C3b, and C3d–C9 [184]. Moreover, increased levels of other molecules have also been reported in the vitreous of diabetic patients with PDR, including prothrombin, alpha 1-antitrypsin, antithrombin III, and factor XIII [185]. RAGE is an innate immune pattern recognition receptor that interacts with AGE to initiate inflammatory responses. A previous study indicates that RAGE levels are increased in DR [186]. The study also showed that RAGE inhibitors can block the inflammatory response induced by hyperglycemia and suppress ICAM-1 expression induced by diabetes [187]. Eicosanoids are signaling molecules produced by oxidation of fatty acids. They participate in inflammatory immune responses and relay messages in the central nervous system. The two main families of eicosanoids are prostaglandins and leukotrienes. Increased induction of COX and prostaglandin production was reported in the retina of diabetic animals [188]. Inhibition of COX reduced the upregulation of VEGF in the retina, as well as vascular permeability and leukostasis [188, 189]. The production of leukotrienes is also increased in the retina of diabetic patients [190], and inhibition of these molecules reduces the degeneration of retinal capillaries and leukostasis [191].

## 12. Conclusions

Inflammation plays an important role in DR pathogenesis and, according to Adamis [45], DR may be considered as a low-grade inflammatory disease. This hypothesis is supported by both experimental and clinical evidence. In animals, proinflammatory cytokine levels are increased in the retina of diabetic animals, and inhibition of TNF-*α* exerts beneficial effects in the prevention of early diabetic retinopathy [50]. Clinical studies have shown elevated levels of proinflammatory cytokines in the vitreous fluid of patients with proliferative diabetic retinopathy, which is related to the severity and progression of retinal injury. Altogether, this evidence suggests that inflammation and ocular diabetic complications are linked and that the levels of circulating inflammatory factors may predict the onset and progression of diabetic retinopathy [6, 10, 12, 13, 31, 192].

Although vascular endothelial growth factor (VEGF) levels were found to be significantly increased in ocular tissues from patients with diabetes [193], the potential role of VEGF in the pathogenesis of DR is not yet completely understood. In fact, a significant decrease of VEGF levels in the aqueous humor occurs after intravitreal injection of anti-VEGF compounds; however, the VEGF levels remain higher than those found in control subjects. Thus, the improvement following anti-VEGF treatment cannot be wholly attributed to the antiangiogenic effect [13]. Nakao et al. recently demonstrated that intravitreal injection of anti-VEGF inhibits leukocyte trafficking in the retina, suggesting that anti-VEGF therapy also acts on retinal inflammation [194]. In contrast, the Early Treatment Diabetic Retinopathy Study demonstrated that the incidence of DR was reduced in human patients taking salicylates for rheumatoid arthritis [193], further confirming the close link between microvascular complication and inflammation in the pathogenesis of diabetic retinopathy. Even these anecdotal findings appear to indicate that treatment with anti-inflammatory compounds along with antineovascularization agents may exert beneficial actions on diabetic ocular complications and in the near future could be translated into clinical practice.

## Figures and Tables

**Table 1:** Mechanisms influencing inflammatory responses in diabetes.

Factor	Function
Apoptotic cell death	Alteration of retinal structures and stimulation of the release of inflammatory mediators

Hyperglycemia	Upregulation of inflammatory molecule expression, promoting leukostasis, and increasing vascular permeability

Oxidative stress	Increasing inflammation and vascular permeability

Metabolism of fatty acids	Inducing inflammatory responses in the retina

AGE/RAGE interaction	Enhancing retinal inflammation

Hypertension	Increasing inflammation through the expression of retinal VEGF and ICAM-1

**Table 2:** Vitreous mediators involved in the pathogenesis of DR.

Vitreous mediators		Function
Cytokines	IL-6	(i) Regulating immune responses (ii) Increasing vascular permeability (iii) Angiogenesis (iv) Regulating expression of metalloproteinases
	IL-8	(i) Chemoattractant (ii) Angiogenesis
	IL-1*β*	(i) Angiogenesis (ii) Synthesizing collagen
	TNF-*α*	(i) Antiangiogenic activity, but also proangiogenic effects under certain conditions (ii) Increasing retinal endothelial permeability (iii) Leukocyte adhesion (iv) Oxidation
	HMGB1	(i) Stabilizing the formation of nucleosomes and gene transcription (ii) Attenuating retinal injury after ischemia-reperfusion (iii) Mediating the secretion of survival factors

Transcription factors	NF-*κ*B	(i) Regulating immune response, cell proliferation, and apoptosis (ii) Synthesizing cytokines, chemokines, and proinflammatory molecules
	HIF-1	(i) Regulating cellular responses under acute and chronic hypoxic condition (ii) Regulating VEGF expression

Chemokines	MCP-1	(i) Recruiting and activating macrophages (ii) Fibrosis and angiogenesis
	IP-10	Inhibiting angiogenesis
	MIG	Angiostatic activity
	SDF-1	(i) Stimulating the mobilization of cells involved in tissue repair and promoting migration, proliferation, and differentiation of endothelial progenitor cells (ii) Promoting repair after ischemic injury (iii) Angiogenesis
	Fractalkine	Angiogenesis
	MIF	(i) Recruiting macrophages to the sites of inflammation (ii) Enhancing macrophages adherence, motility, and phagocytosis

Growth factors	VEGF	(i) Increasing vascular permeability (ii) Angiogenesis (iii) Endothelial cell migration and survival (iv) Expression of ICAM and VCAM-1
	PGF	(i) Potentiating the action of VEGF (ii) Stimulating endothelial cell proliferation, migration, and angiogenesis
	Tenascin-C	(i) Modulating cell growth and cell adhesion (ii) Involved in sprouting of endothelial cells
	IGF 1	(i) Regulating the proliferation and differentiation of several cell types (ii) Stimulating the production of VEGF
	bFGR	(i) Survival/maturation of neurons and glial cells (ii) Angiogenesis
	HGF	(i) Modulating the motility, growth, and morphogenesis of various cell types (ii) Angiogenesis
	NGF	Stimulating Muller cells to produce bFGF, which in turn stimulates endothelial cell proliferation and secretion of VEGF
	CTGF	Stimulating proliferation, angiogenesis, migration, extracellular matrix production, cell attachment, cell survival, and apoptosis
	Stem cell factor	(i) Involved in survival and differentiation of hematopoietic stem cells (ii) Promoting survival, migration, differentiation, and capillary tube formation of endothelial cells
	EPO	Antioxidant, anti-inflammatory, proangiogenic, neuroprotective, and antiapoptotic properties
	Adiponectin	Anti-inflammatory and antiatherosclerotic properties

Adhesion molecules	ICAM-1, VCAM-1, and E-selectin	Recruiting leukocytes
	Soluble vascular adhesion protein	Recruiting leukocytes

## Abbreviations

AGE: Advanced glycation end products
APN: Aminopeptidase N
bFGF: Basic fibroblast growth factor
COX: Cyclooxygenase
CTGF: Connective tissue growth factor
DME: Diabetic macular edema
DR: Diabetic retinopathy
EPO: Erythropoietin
HIF-1: Hypoxia-inducible factor-1
HGF: Hepatocyte growth factor
HMGB1: High-mobility group box protein-1
ICAM-1: Intercellular adhesion molecule-1
IGF-1: Insulin-like growth factor-1
IL: Interleukin
IP-10: Interferon-gamma inducible protein 10
MCP-1: Monocyte chemotactic protein-1
MIF: Macrophage migration inhibitory factor
MIG: Monokine induced by interferon-*γ*
MMPs: Matrix metalloproteinases
NF-*κ*B: Nuclear factor-kappa B
PDR: Proliferative diabetic retinopathy
PGF: Placental growth factor
PKC: Protein kinase C
RAGE: Receptor for advanced glycation end products
RANKL: Receptor activator of nuclear factor-kappa B
TNF-*α*: Tumor necrosis factor-alpha
SDF-1: Stromal cell-derived factor-1
VCAM: Vascular cell adhesion molecule-1
VEGF: Vascular endothelial growth factor.
